# Supplementary figures and images for: Improving basic and translational science by accounting for litter-to-litter variation in animal models
Source: BMC Neurosci. 2013 Mar 22;14:37. doi: 10.1186/1471-2202-14-37 (PMC3661356; doi:10.1186/1471-2202-14-37)

**A**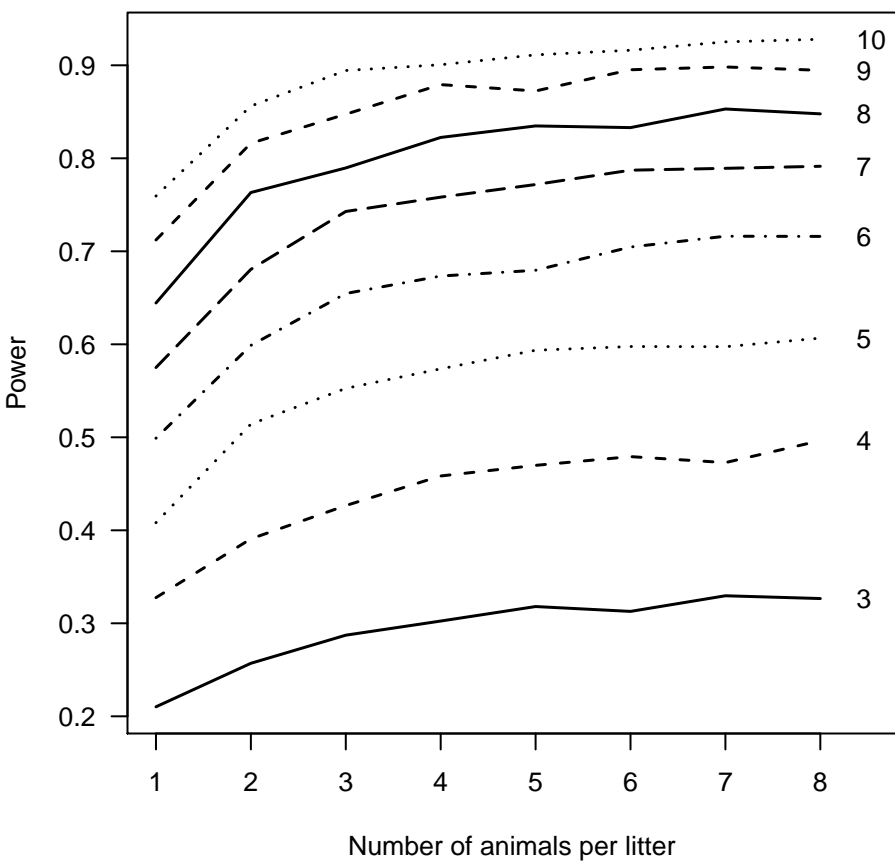**B**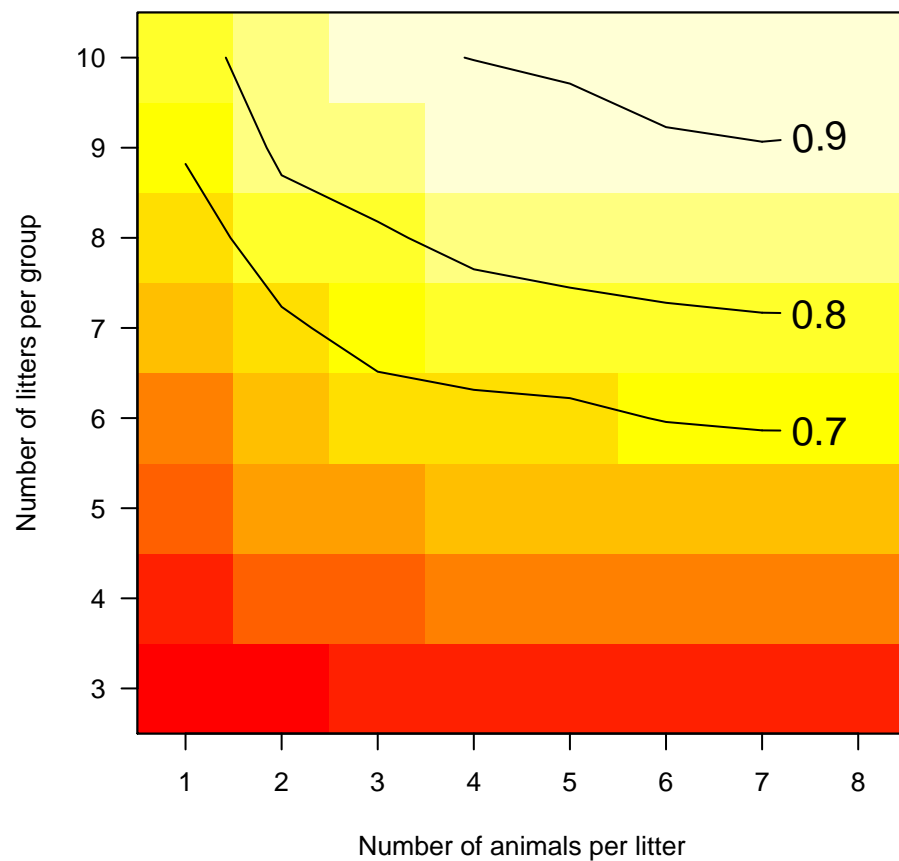**C**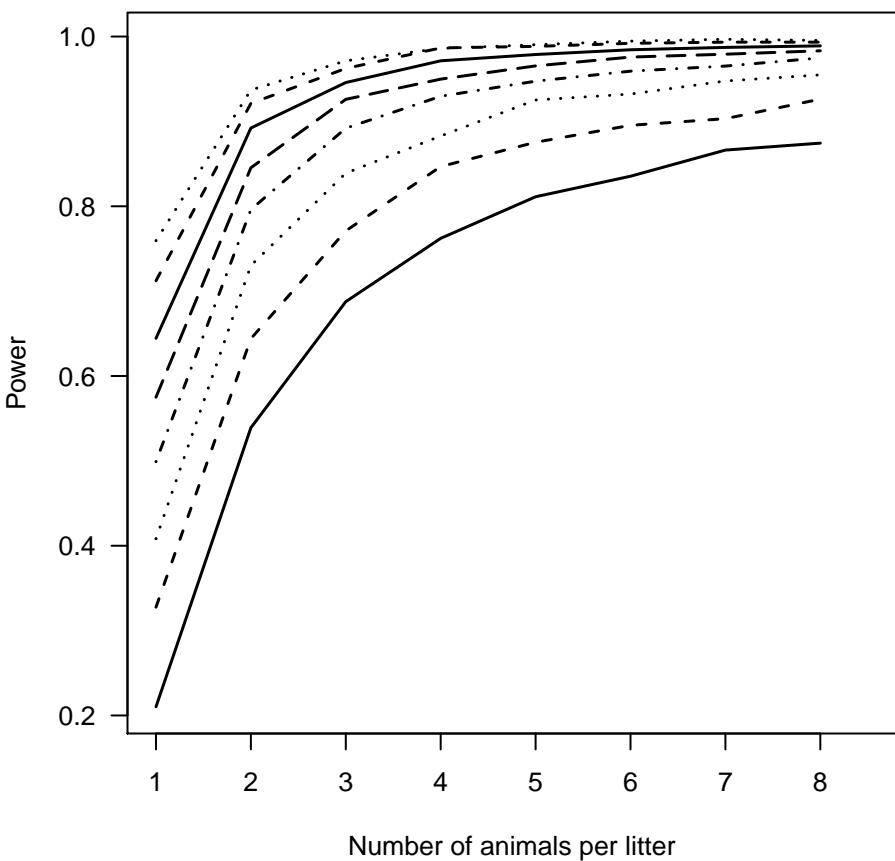**D**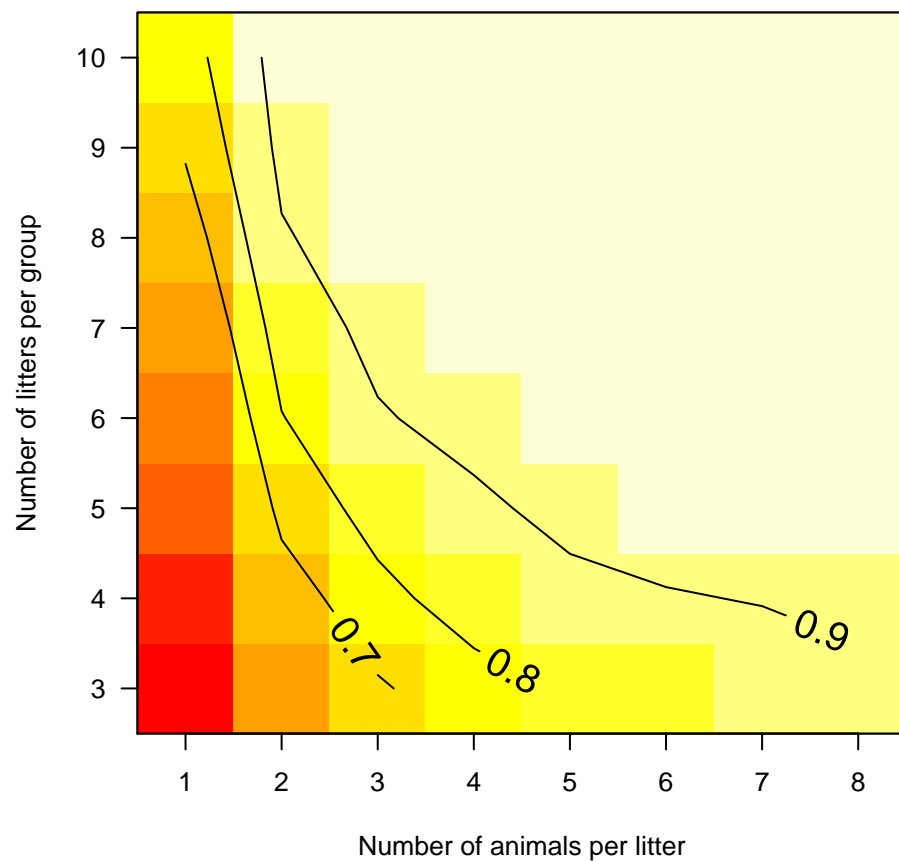

Supplement: Additional file 4 — Power analysis for the mixed-effects model and the incorrect analysis. The interpretation of the graphs is the same as Figure 4 (main text). Panels A and B are for the mixed-effects model and are nearly identical to the results for averaging the values within each litter and then using a t-test (Figure 4 main text). Panels C and D ignore litter and compare all of the data with a t-test, which results in an artificially inflated sample size and inappropriately high power. [file 1471-2202-14-37-S4.pdf]
